# Supplementary material for: A Critical View of the Application of the APEX Software (Aqueous Photochemistry of Environmentally-Occurring Xenobiotics) to Predict Photoreaction Kinetics in Surface Freshwaters
Source: Molecules. 2019 Dec 18;25(1):9. doi: 10.3390/molecules25010009 (PMC7017383; doi:10.3390/molecules25010009)
Supplement: Supplementary file 1 [file molecules-25-00009-s001.zip › 3.2.4_gcc-4.4.0.zip!/3.2.4_gcc-4.4.0/3.2.4_gcc-4.4.0/doc/octave/html/interpreter/Data-Structure-Objects.html]

Data Structure Objects - Untitled


Next: Cell Array Objects,
Previous: String Objects,
Up: Built-in Data Types


---

#### 3.1.4 Data Structure Objects

Octave's data structure type can help you to organize related objects of
different types. The current implementation uses an associative array
with indices limited to strings, but the syntax is more like C-style
structures.

See Data Structures, for more information.
